# Impact of incomplete lineage sorting and natural selection on the phylogenetic and taxonomic uncertainties of *Aspidistra* in Taiwan

**DOI:** 10.1186/s40529-025-00482-y

**Published:** 2025-10-15

**Authors:** Min-Xin Luo, Ming-Jen Yang, Chang-Tse Lu, Pei-Chun Liao

**Affiliations:** 1 School of Life Science, National Taiwan Normal University, No. 88 Ting-Chow Rd., Sec. 4, Taipei, 116 Taiwan; 2 Department of Ecology and Evolutionary Biology, University of Michigan, Ann Arbor, MI 48109-1085 USA; 3 Department of Biological Resources, National Chiayi University, 300 Syuefu Rd., Chiayi City, 60004 Taiwan

**Keywords:** *Aspidistra*, Transcriptome, Approximate bayesian computation, Phylogenetic signal, Topological test

## Abstract

**Background:**

The inconsistency between morphological and genetic evidence has consistently presented a challenge in taxonomy. The genus *Aspidistra* (Asparagaceae) comprises perennial herbs found primarily in eastern and southeastern Asia. This group has received limited systematic study despite the continuous publications of new species in recent years. Most species of *Aspidistra* have narrow distributions, and a large number of them are endemic. This study examined five *Aspidistra* species in Taiwan, which are part of a total of around 200 *Aspidistra* species found throughout Asia. In this study, we sampled all *Aspidistra* taxa in Taiwan to explore their phylogenetic relationships. We utilized transcriptome data for phylogenetic reconstruction and employed gene genealogy interrogation (GGI) to identify conflicts between gene trees and the species tree. Additionally, we tested nine evolutionary scenarios for these taxa by incorporating population-level genetic data. We obtained a well-supported species tree but also detected a high proportion of incomplete lineage sorting (ILS) phenomena.

**Results:**

The results revealed that the two varieties of *A. daibuensis* failed to form monophyly despite morphological similarities. However, about 20.8% of the genes did not reject the topology that grouped them together. Among these genes, we identified positive signals in photosynthesis-related genes, suggesting their similarities arose from convergent evolution. Furthermore, we used a phylogenetic signal test to identify the evolutionary meaningful traits and found that the stigma width can reflect the phylogenetic relationships among these species.

**Conclusions:**

Our study provides new insights into the evolutionary dynamics and taxonomy of *Aspidistra* in Taiwan, revealing key genetic and morphological patterns shaping species divergence. Phylogenetic analysis revealed substantial ILS, with numerous genes supporting alternative tree topologies. Despite morphological similarities, *A. daibuensis* var. *daibuensis* and var. *longkiauensis* exhibit non-monophyletic relationships, challenging their previous classification. Genes associated with chloroplastic function and photomorphogenic adaptation suggest convergent evolution. Moreover, stigma shape emerges as a robust diagnostic trait for species delimitation. These findings underscore the complex interplay of genetics, morphology, and adaptation, emphasizing the need for further integrative research.

**Supplementary Information:**

The online version contains supplementary material available at 10.1186/s40529-025-00482-y.

## Background

In phylogenomic studies, gene tree-species tree incongruence can occur due to incomplete lineage sorting (ILS) and introgression, which operate through separate mechanisms. Incomplete lineage sorting happens when alleles within populations fail to coalesce due to the retention of ancestral polymorphisms. This can lead to hemiplasy (Avise, Robinson 2008), resulting in gene trees that do not align with the species tree. Incomplete lineage sorting is more common in large populations with short speciation intervals (Maddison, Knowles 2006) and is prevalent in sexually reproducing organisms, especially recent divergences (Steenwyk et al. 2023).

On the other hand, introgression occurs during hybridization when genetic material is exchanged between species, introducing alleles with evolutionary histories that differ from the species tree (Hibbins and Hahn 2022). Detecting introgression can be challenging, especially for large genomic regions, and methods like the *D*-statistic (Durand et al. 2011; Pease and Hahn 2015) and branch-length tests (Hahn, Hibbins 2019) are used. Both ILS and introgression contribute to gene tree-species tree incongruence, but they are distinct evolutionary processes that have unique implications for understanding the evolutionary history of organisms.

Natural selection is another factor that may influence phylogenetic conflicts (Steenwyk et al. 2023). The influence of natural selection on molecular evolution can introduce complexities into phylogenetic analyses. When positive selection leads to convergent evolution of sequences, it can mislead phylogenetic reconstructions by grouping sequences with similar traits due to selection rather than common ancestry (Sackton and Clark 2019). Accurately unraveling the evolutionary relationships among species and genes requires understanding the potential effects of natural selection on phylogenetic inference.

Although using genome-scale data for molecular phylogenies has potential benefits, recent studies have highlighted conflicts in large-scale phylogenomics (Steenwyk et al. 2023). The main challenge is that current methods may not be suitable for processing vast data, leading to systematic biases and inconsistent clade support (Philippe et al. 2011; Reddy et al. 2017; Salichos and Rokas 2013). Additionally, errors may arise from poor taxonomic sampling, varied gene orthology criteria, and complex interactions among these factors. These issues can be reduced by filtering datasets and focusing on informative loci, but incongruences may still persist (Reddy et al. 2017). As a result, hypothesis-testing procedures tailored for phylogenomics, such as gene genealogy interrogation (GGI), have become increasingly important (Betancur-R et al. 2019).

*Aspidistra* Ker Gawl. (Asparagaceae) is a subtropical to tropical genus, mainly distributed in eastern and southeastern Asia, comprising more than 200 species (Kalyuzhny et al. 2022). Species belonging to this genus are usually found in lowlands below an altitude of 1500 m (Tillich 2014). Most species have limited ranges; some are exclusively found in a single location. They grow in the understory of forests and usually flower on the ground, often concealed by litter material. As a result, new species are continuously being discovered and described.

The plant body of *Aspidistra* species is composed of a vertical rhizome with alternate leaves. The flowers are solitary or multiple and are located near the terminal nodes of the rhizome. The most crucial characteristic of *Aspidistra* is the flower structure, particularly the stigma, which varies greatly (Li 2004; Tillich 2014). An infragenus classification system for *Aspidistra* was proposed by Li (2004) based on stigma morphology, but this system was not supported by phylogenetic studies (Huang et al. 2013). Although most species have been described based on morphology, molecular biological methods have rarely been used to discuss *Aspidistra* species.

Taiwan, a continental island situated southeast off mainland Asia, harbours five taxa (three species and two varieties), including *A. attenuata* Hayata, *A. daibuensis* Hayata var. *daibuensis*, *A. daibuensis* Hayata var. *longkiauensis* C.T.Lu, Ming Jen Yang & J.C.Wang, *A. mushaensis* Hayata var. *mushaensis*, and *A. mushaensis* Hayata var. *longiconnectiva* (C.T.Lu, K.C.Chuang & J.C.Wang) C.T.Lu & J.C.Wang (Lu et al. 2020, 2023). These five taxa are all endemic to Taiwan. Due to morphological and genetic similarity, *A. longiconnectiva* (Lu et al. 2020) was recently reduced to *A. mushaensis* var. *longiconnectiva* (Lu et al. 2023). Another variety, *A. daibuensis* var. *longkiauensis*, is genetically distinguishable, revealed by simple-sequence repeats (SSR), despite similar morphology (Lu et al. 2023). The consolidation or separation of taxa, although only changes in rank, can affect evolutionary inferences and conservation applications. Therefore, it is necessary to provide more evidence to reexamine their taxonomic ranks.

When examining the evolutionary history of a species, it is expected that the different varieties of a species will be grouped in a monophyletic manner. The genetic separation of two varieties of *A. daibuensis* (Lu et al. 2023) may lead to two hypotheses: (1) var. *daibuensis* and var. *longkiauensis* are reproductively isolated and monophyletic, or (2) they have convergent phenotypes and are non-monophyletic. If the latter is true, these two taxa cannot be classified within a single species. However, the change in the taxonomic rank of Taiwanese *Aspidistra* has yet to be verified through phylogenetic analysis. Furthermore, *A. mushaensis* var. *mushaensis* and var. *longiconnectiva* have mixed genetic components from *A. daibuensis* var. *longkiauensis* and *A. attenuata* (Lu et al. 2023), implying a possibility of a hybrid origin of *A. mushaensis*.

Lu et al. (2023) conducted morphological analyses on Taiwanese *Aspidistra* and found minor genetic inconsistencies. The environment can influence many morphological characteristics, particularly vegetative organs (Shimizu-Inatsugi et al. 2023). While some traits may help identify differences between certain species, they may not apply to other taxa due to their lack of analytical power in reflecting phylogenetic relationships and evolutionary rates. On the other hand, phylogenetically conservative characteristics can provide more accurate information about evolutionary relationships and species divergence rates, making them better suited for systematic classification (Zhang et al. 2016). With this in mind, we will revisit Lu et al.‘s (2023) research to identify the most reliable diagnostic characteristics of *Aspidistra* in Taiwan.

In this study, we specifically conducted phylogenetic analysis on five taxa of *Aspidistra* from Taiwan. This study examined the taxonomic challenges and evolutionary relationships of the *Aspidistra* species in Taiwan. We employed both genomic analysis and morphological studies to clarify the taxonomic relationships among these species, which have previously been obscured by unclear classifications in Taiwan. Our research, which builds upon the findings of Lu et al. (2023), specifically examines the relationship between *A. daibuensis* var. *daibuensis* and *A. daibuensis* var. *longkiauensis*. Our analysis of thousands of genes obtained from transcriptomes highlights the impact of ILS, introgression, and positive selection on the conflict between gene trees and the species tree of our study subjects.

## Materials and methods

### Sampling and data collection

We collected nine samples from the field, encompassing five taxa in Taiwan. The plants collected were planted in a common garden, and fresh tissues were collected from the young shoots or root apical meristems for RNA extraction. Six species, *A. zongbayi*, *A. elatior*, *A. fenghuangensis*, *Tupistra fungilliformis*, *Reineckea carnea*, and *Rohdea japonica* were used as outgroups. The detailed information is listed in Table S1.

### RNA extraction

Total RNA was extracted from plant powder using the modified CTAB method with NaCl and PVPP to remove polysaccharides and polyphenols. The extraction buffer contained 2% CTAB, 2% PVPP, 2 M NaCl, 100 mM Tris-base, 20 mM EDTA, pH 7.5, and 2% of β-mercaptoethanol. After heating and centrifuging, the supernatant was mixed with acid phenol-chloroform and centrifuged again. The aqueous phase was transferred to another tube with isopropanol and LiCl, and the mixture was centrifuged again before washing the pellet with 70% ethanol and resuspending in DEPC water.

### RNA sequencing, filtering, and de novo assembly

The RNA samples were sequenced by Illumina NovaSeq 6000 platform with 150 bp pair-end sequencing at Genomics, Taiwan. Transcriptome files of *A. fenghuangensis* were downloaded from NCBI and converted to fastq format using the SRA toolkit. Data quality was assessed using FastQC v0.11.9 (Andrews 2010) and MultiQC v1.10 (Ewels et al. 2016). We removed adapters and low-quality reads by Trimmomatic v0.39 (Bolger et al. 2014). We performed de novo assembly using Trinity v2.12 (Grabherr et al. 2011) and removed redundant transcripts (> 99% similarity) using CD-HIT v4.8.1 (Fu et al. 2012; Li and Godzik 2006). TransDecoder v5.5.0 (Haas 2013) and OrthoFinder v2.5.4 (Emms and Kelly 2019) were used to predict genes and infer orthologs, respectively.

### RNAseq dataset analyses

#### Species tree reconstruction

From orthologs inference, we obtained 1,029 single-copy genes. ASTRAL-II v5.7.7 (Mirarab and Warnow 2015) provides a method to infer the species tree from gene trees using a coalescent-based approach. To prepare for ASTRAL-II input, we constructed maximum likelihood gene trees by IQ-TREE v2.1.2 (Minh et al. 2020; Nguyen et al. 2015). The species tree was then examined with the “-q” option to determine the normalized quartet score and the “-t 3” option, providing the local posterior probability for each branch in ASTRAL-II.

We employed Bayesian multispecies coalescent (MSC) analysis to estimate species tree and molecular dating with a strick clock model in StarBEAST3 v1.0.5 (Bouckaert et al. 2019; Douglas et al. 2022). The prior clock rate was set at 0.005/site/million years, referring to the average rate of silent-site divergence (*µ*) in angiosperms (De La Torre et al. 2017). We retained a strict clock with a fixed substitution rate in StarBEAST3 because our focus was on relative divergence times among closely related Taiwanese taxa, for which a well-established angiosperm-wide average rate is appropriate. Due to the lack of suitable fossil evidence for calibration points and the limited analysis of 189 genes, the relaxed clock model’s effectiveness is restricted. Thus, we used a strict clock for preliminary computations. To address rate variation more fully, we complemented this approach with MCMCTree analyses using a relaxed-clock prior (see below). Due to the Bayesian Markov chain Monte Carlo being a time-consuming task, we only used the 189 genes whose topology aligns with the result of ASTRAL estimating by topological test (see below). For each gene, we used Jmodeltest v2.1.10 (Darriba et al. 2012; Guindon and Gascuel 2003) to select the best-fit substitution model according to BIC. Analyses were run for 100,000,000 Markov Chain Monte Carlo (MCMC) generations, sampling every 25,000th generation. We used Tracer v1.7 (Rambaut et al. 2018) to check the effective sample size (ESS) of parameters and discard 10% of the trees as burn-in. DensiTree (Bouckaert 2010) was used to visualize the posterior probability distributions of trees.

Given that *Aspidistra* represents a rapidly radiating lineage, substitution rate heterogeneity may bias molecular dating. To improve temporal calibration, we included outgroup species from closely related genera. Four secondary calibration points, based on Ji et al. (2022), were applied. For divergence time estimation, we identified 341 single-copy genes from 15 samples representing 11 operational taxonomic units (OTUs). Species tree topology was inferred using ASTRAL-II, followed by divergence time estimation using MCMCTree (Rannala and Yang 2007; Reis, Yang 2011). For OTUs represented by multiple samples, we generated consensus sequences using the most frequent base at each position, implemented with the R package Biostrings (Pagès et al. 2025). In MCMCTree, we implemented a relaxed-clock model with a lognormal prior on substitution rates, which allows for among-lineage rate variation. For the overall substitution rate, we estimated from the sequences by Baseml in PAML. We specified a diffuse gamma prior (G(1, 12.5), corresponding to Gamma (*α* = 1, *β* = 12.5) with a mean of 0.08 substitution per site per year, in order to avoid overly restrictive assumptions. This setting follows recommendations for datasets with potential rate heterogeneity. Two independent MCMC runs were conducted, each for two million generations with samples taken every 1,000 iterations, resulting in 20,000 samples per run. Convergence was assessed by comparing the posterior mean node ages between the two runs (Figure S1, and prior versus posterior distributions of calibrated node ages were evaluated (Figure S2).

#### Topological test

In order to comprehend phylogenetic conflicts, we utilized IQ-TREE v2.1.2 (Minh et al. 2020; Nguyen et al. 2015) to conduct a topological test. The topological test is the GGI strategy (Arcila et al. 2017). Our topological test focused on two key inquiries: (1) how many gene trees fit the *dai-lgk* morphological resemblance? Moreover, (2) how many gene trees fit the species trees reconstructed by ASTRAL-II? We combined the variety *lcn* into *mus* and tested the remaining four taxa for 15 hypothetical tree topologies for each gene (Fig. 3). Samples of the same species were constrained within a clade. The tests were evaluated using an approximately unbiased (AU) test (Shimodaira 2002) with 10,000 resamplings of estimated log-likelihoods (RELL) replicates (Kishino et al. 1990) in IQ-TREE. It is imperative to note that we selected the best-fit tree topology for each gene with the lowest ΔL value. Gene annotation was conducted for those genes that fit the *dai-lgk* morphological resemblance.

#### Positive selection

The positive selection was tested for 214 genes (see results) that did not reject the hypothesis of *dai-lgk* morphological resemblance. The branch model of PAML was conducted with a priori of the ASTRAL species tree and the *dai* and *lgk* lineages as foregrounds. The constant model was set as the null model. The likelihood ratio test assessed the significance of positive selection (*Ka/Ks* > 1).

#### Phylogenetic signals

We calculated the phylogenetic signal of 21 morphological traits measured by Lu et al. (2023) with Blomberg’s *K* (Blomberg et al. 2003) within R package geiger (Harmon et al. 2009). The species tree, reconstructed by StarBEAST, served as the input tree. We employed the Brownian model as the null model and conducted 10,000 simulations for a randomization test.

#### Gene annotation

We used MACSE v2 (Ranwez et al. 2011) to align the nucleotide sequences and translate them to amino acid sequences. Subsequently, BLASTP v2.11.0+ (Camacho et al. 2009) was used to compare amino acid sequences to the *Arabidopsis* Information Resource (TAIR) database (Berardini et al. 2015) with a threshold E-value of 1e-6.

### SSR dataset analyses

#### STRUCTURE analysis

Lu et al. (2023) conducted a STRUCTURE analysis but only presented *K* = 2 and *K* = 4 without showing the hierarchical genetic structure. We replicated their analysis with their SSR data and presented the hierarchical STRUCTURE result (*K* = 2 ~ 6).

#### Network analysis

The SSR data was transformed to a distance tri-matrix and output nexus format using GenAlex v6.503 (Peakall and Smouse 2006) to reconstruct the network employing the NeighborNet algorithms within SplitsTree v4.14.4 (Huson and Bryant 2006). The output files generated by SplitsTree were then visualized using the R package ggnetworx (Paradis and Schliep 2019; Schliep et al. 2017; Schliep 2011; Yu et al. 2017).

#### Principal component analysis

The principal component analysis (PCA) was conducted by R package adegenet (Jombart 2008) and visualized by R package ggplot2 (Wickham et al. 2016).

#### Approximate bayesian computation (ABC)

According to the STRUCTURE analysis, two distinct groups, *dai-mus* and *att-lgk*, emerge when *K* = 2. However, at *K* = 4, the genetic resemblance of *att-lgk* disappears, while the *dai-mus* genetic cluster continues to persist. The *dai-mus* genetic cluster was also evident in the NeighborNet analysis. This observation leads to the formulation of a hypothesis suggesting a genetic resemblance between *dai* and *mus*. Additionally, when *K* = 4, it becomes evident that *mus* originates as a hybrid of *dai* and certain components of *att*, implying a hybridization scenario. Considering morphological characteristics, *lgk* resembles *dai*, classifying these two taxa as varieties of *A. daibuensis*. The species tree reconstruction, utilizing 1,029 genes with the MSC method, proposes a hypothesis wherein *att* and *lgk* share genetic similarities with *mus* (including its variety *lcn*), while *dai* shows a more distant relationship (see Results). Based on these findings, four plausible hypotheses are postulated:

##### Hypothesis 1

*mus* originated through hybridization between *att* and *dai* (hybrid scenarios: **a** and **b**).

##### Hypothesis 2

The species tree hypothesis suggests (((*att*, *lgk*), *mus*), *dai*) (1029-genes species tree scenario: **c**).

##### Hypothesis 3

*dai* exhibits greater similarity to mus than to *lgk* (*dai-mus* genetic resemblance scenarios: **d**, **e**, and **f**).

##### Hypothesis 4

*dai* and *lgk* form a monophyletic group (*dai-lgk* morphological resemblance scenarios: **g**, **h**, and **i**).

In total, nine evolutionary hypotheses were compared using Approximate Bayesian Computation (ABC). The summary statistics of observed data were computed using arlsumstat v3.5.2 (Excoffier and Lischer 2010). For each model, one million simulations were performed by fastsimcoal26 (Excoffier, Foll 2011; Excoffier et al. 2013). After simulations, we removed the highly correlated summary statistics with a Pearson correlation coefficient |*r*| > 0.8. The following model selection and parameter estimation were performed using the R package abc (Csilléry et al. 2012). Out of one million simulations, subsets of five thousand were retained for model selection, and the model with the highest posterior probability was considered the best model. The parameters were log-transformed and estimated from a subset of two thousand simulations.

## Results

### Species tree reconstruction and topological tests

**Fig. 1:**
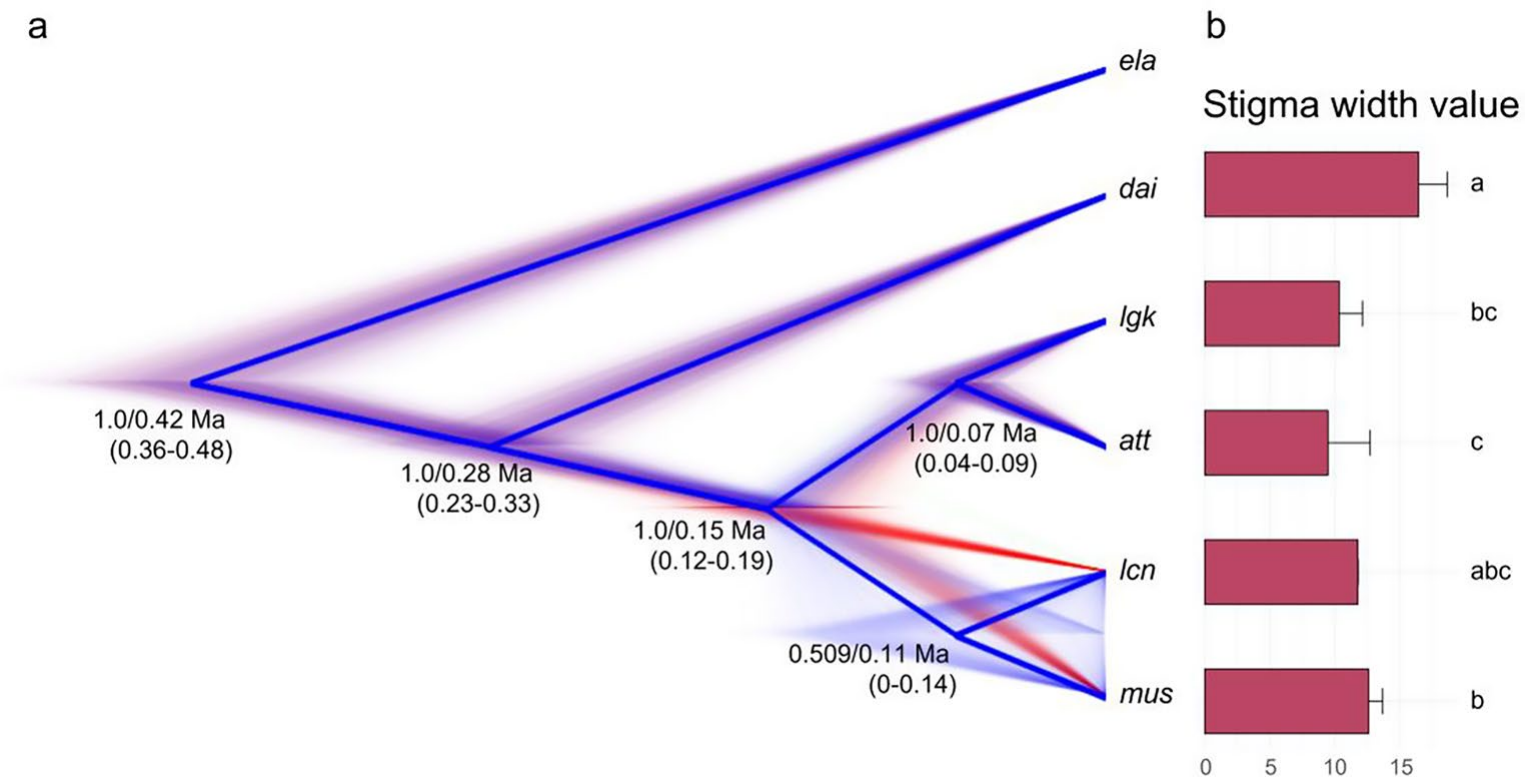
(**a**) Species tree topology and molecular dating reconstructed by StarBEAST3 with 189 genes and (**b**) stigma width value of five taxa in Taiwan. The number on the node represents the supporting value/median value of molecular dating, and the number in the parentheses is the 95% confidence interval of molecular dating. The confusion between *lcn* and *mus* on DensiTree and their low supporting value shows they may not be completely differentiated

**Fig. 2:**
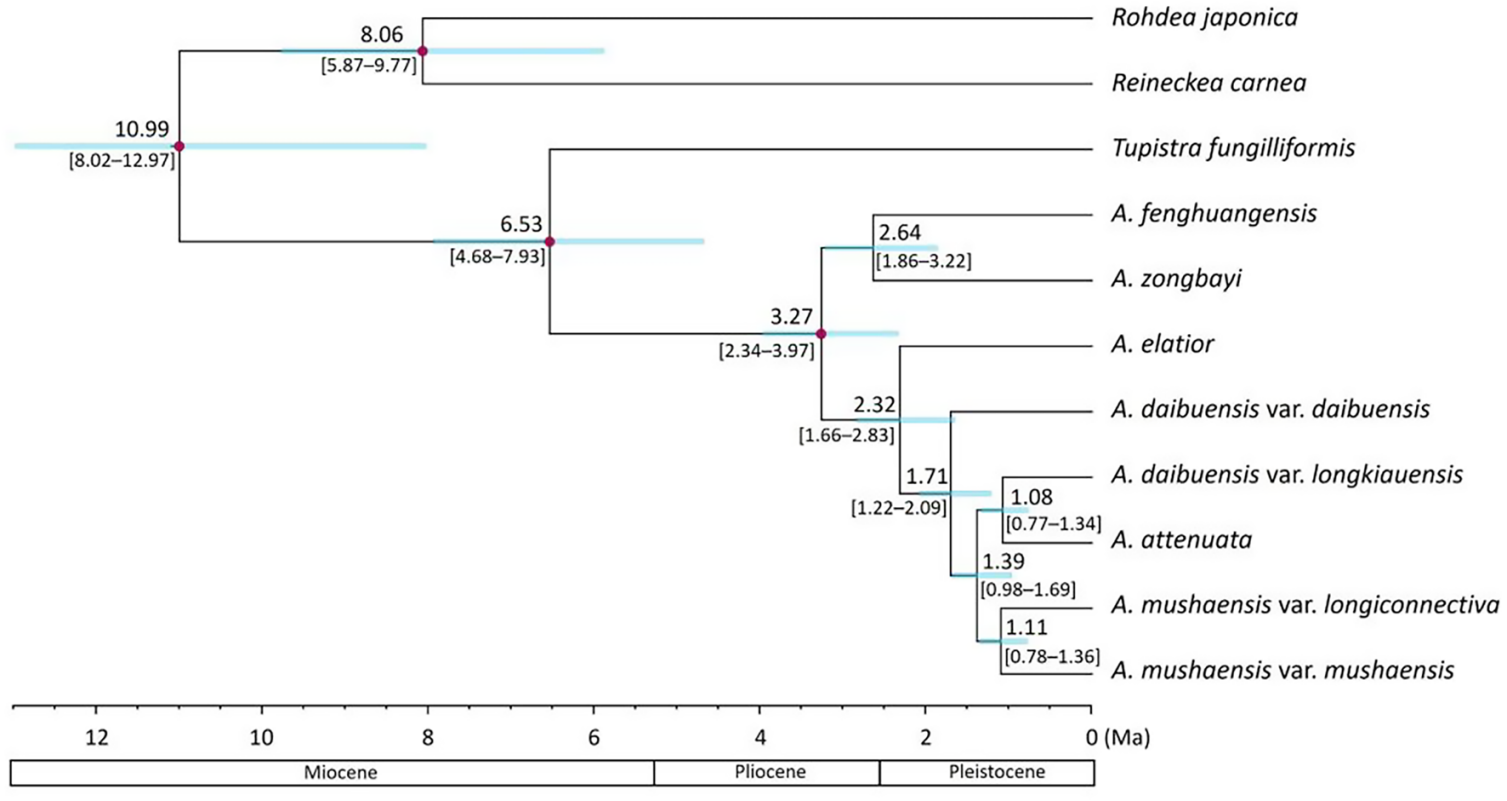
Species tree inferred using ASTRAL, with divergence times estimated by MCMCTree. Red dots indicate the four secondary calibration points. Blue bars at the nodes represent the 95% credibility intervals of divergence time estimatess

The species tree reconstructed by ASTRAL with 1,029 genes and by the MSC method with 189 genes showed *att*-*lgk* and *mus*-*lcn* as sister groups, while *dai* appeared more distantly related (Fig. S3 and 1a). This tree topology supports the taxonomic treatment of *mus* (var. *mushaensis*) and *lcn* (var. *longiconnectiva*) within a species (*A. mushaensis*) but suggests *lgk* (var. *longkiauensis*) should not be a variety of *A. daibuensis*. The molecular dating indicates that the Taiwanese *Aspidistra* diverged from its sister *A. elatior* at 0.42 Ma (95% confidence interval (CI): 0.36–0.48 Ma). The divergence between *mus* and *lcn* has relatively low supporting value (0.509), suggesting their incomplete differentiation (Fig. 1a). The relationships were also recovered in the tree calibrated using secondary points; however, the estimated divergence times were older with the crown age of *Aspidistra* was at 3.27 Ma (95%CI: 2.34–3.97 Ma) and the origin of the Taiwanese *Aspidistra* lineage at 1.71 Ma (95%CI: 1.22–2.09 Ma) (Fig. 2).

**Fig. 3:**
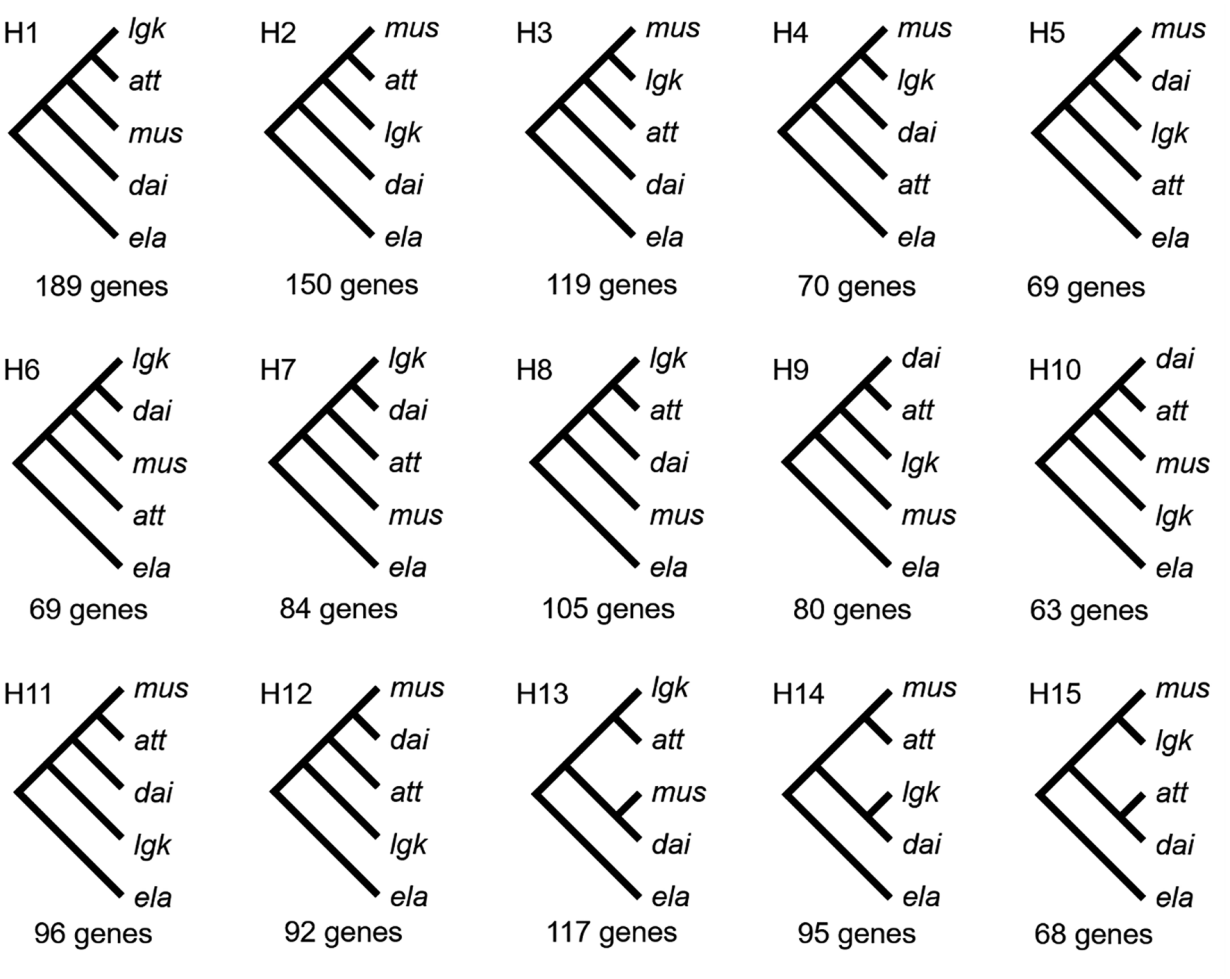
The summary of the AU test results includes 15 hypothetical tree topologies, along with the number of genes in each tree that were unable to reject the topology. It’s worth noting that a single gene may not reject just one tree topology, leading to the sum of genes (1466) exceeding 1029. Abbreviations *att*, *dai*, *lgk*, *mus*, and *ela* denote *A. attenuate*, *A. daibuensis* var. *daibuensis*, *A. daibuensis* var. *longkiauensis*, *A. mushaensis* (including var. *mushaensis* and var. *longiconnectiva*), and *A. elatior*, respectively

**Fig. 4:**
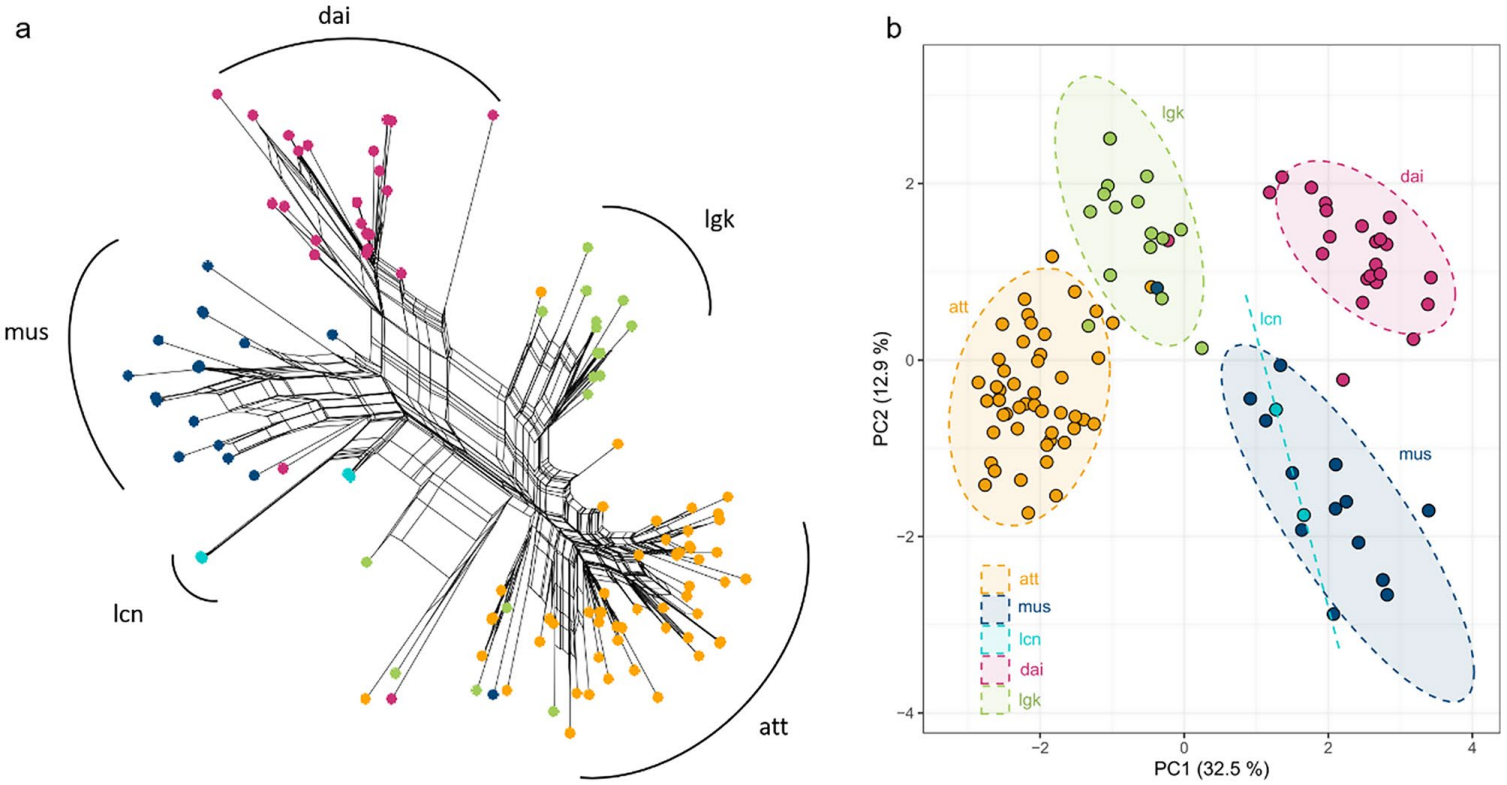
Genetic clustering of *Aspidistra* in Taiwan. (**a**) NeighborNet network; (**b**) Principal component analysis (PCA). Abbreviations *att*, *dai*, *lgk*, *mus*, and *lcn* denote *A. attenuata*, *A. daibuensis* var. *daibuensis*, *A. daibuensis* var. *longkiauensis*, *A. mushaensis* var. *mushaensis*, and *A. mushaensis* var. *longiconnectiva*, respectively

We performed a topological test to determine how many genes support the MSC species tree and merged *lcn* with *mus* to simplify the process. After analyzing 15 different topologies (Fig. 3), we found that the species tree topology was the most supported hypothesis, receiving the fewest rejections from the genes. It was found that 18.37% of genes (189 out of 1029) aligned with the species tree hypothesis (Fig. 3, H1). However, more than 80% of gene trees did not match the species tree topology, suggesting complex reticulate evolutionary connections as revealed by NeighborNet (Fig. 4a). This also suggests the presence of incomplete lineage sorting (ILS), introgression, or selection on these genes.

### dai-lgk resemblance

Since the current taxonomic treatment that *dai* and *lgk* were within a species, we performed the topological test to examine how many genes were consistent with the sister relationship of *dai* and *lgk*. The result shows that 20.80% of genes (214 out of 1029) did not reject the monophyletic relationship between *dai* and *lgk* (69, 84, and 95 gene trees for H6, H7, and H14, respectively). These genes revealed diverse functions (Table S2). Among these genes, 19 were found to be chloroplastic, suggesting that photosynthesis-related functions highly contribute to *dai* and *lgk* grouping. Additionally, at least seven genes are ubiquitin-related genes, including E3 ubiquitin-protein ligase, Light-mediated development protein DET1, Ubiquitin system component Cue protein, Ubiquitin family protein AT2G35360, E3 ubiquitin-protein ligase RNF170-like protein, E2 ubiquitin-conjugating enzyme UBC19, and E3 ubiquitin-protein ligase UPL5 (Table S2). Ubiquitin mediates plant cell signaling, stress responses, immunity, and morphogenesis (Doroodian, Hua 2021; Li and Li 2014; Pokhilko et al. 2011; Wang and Deng 2011). We are uncertain about how these genes resemble the morphology of *dai* and *lgk*, but their responses to the environments may play important roles.

### Positive selection for the dai-lgk resemblance

The PAML branch model observed that the *dai* and *lgk* lineages showed significant positive selection signals (*Ka/Ks* > 1) in four genes. These genes are *EARLY LIGHT-INDUCIBLE PROTEIN* (*ELIP1*), *Iojap* (*Ij*), *PROTEASOME BIOGENESIS-ASSOCIATED CHAPERONE 5* (*PBAC5*), and *Protein arginine N-methyltransferase 1.1* (*PRMT11*). *ELIP1* and *Ij* determine plant color, *PBAC5* encodes chaperone function, and *PRMT11* involves arginine methyltransferase activity.

### ILS and introgression

Among the high proportion of gene trees conflicting with the species tree, most were considered as a consequence of the ILS. The QuIBL analysis suggests that, among 12 triplet tree topologies, only four are detected to have significant ILS + introgression events (ΔBIC: BIC_ILS + intro_ – BIC_ILS only_ < −10 (Edelman et al. 2019), while the others cannot reject the ILS-only hypothesis. In these four ILS + introgression triplet tree topologies, only a few proportions of genes can be detected to involve introgression (1, 4, 6, and 12 genes, Table 1). Compared with hundreds of ILS genes, the proportion of these introgression genes is only < 2% (0.10%~1.17%). This shows that the recent rapid speciation of these Taiwanese *Aspidistra* has left numerous genes unable to be completely sorted.

**Table 1:** Summary statistics of the proportion of ILS and introgression (non-ILS) genes inferred by QuIBL. The results showed that the ILS-only model causes most phylogenetic discordances of gene trees, and only 4, 6, 12, and 1 genes (BIC_ILS + intro_ – BIC_ILS only_ < −10 (Edelman et al. 2019) resulted from introgression under the ILS + introgression model

Topology	Prop_ILS_	Prop_non-ILS_	BIC_ILS + intro_	BIC_ILS only_	ΔBIC	Gene numbers	ILS genes	Introgression genes
((att, dai), lgk)	0.935	0.065	−2646.2	−2661.8	15.6	256	239	-
((lgk, dai), att)	0.932	0.068	−2696.4	−2707.2	10.9	258	240	-
((att, lgk), dai)*	0.915	0.085	−5019.1	−5039.0	19.9	509	466	-
**((att**,** mus)**,** lgk)**	**0.988**	**0.012**	**−3343.9**	**−3249.8**	**−94.1**	**331**	**327**	**4**
((lgk, mus), att)	0.877	0.123	−3144.1	−3169.5	25.5	298	261	-
**((att**,** lgk)**, mus)*	**0.985**	**0.015**	**−4001.1**	**−3976.7**	**−24.4**	**392**	**386**	**6**
((dai, mus), lgk)	0.953	0.047	−3119.0	−3140.4	21.5	309	294	-
((lgk, mus), dai)*	0.952	0.048	−4455.5	−4467.3	11.8	445	424	-
**((dai**,** lgk)**,** mus)**	**0.957**	**0.043**	**−2805.3**	**−2788.7**	**−16.6**	**274**	**262**	**12**
((dai, mus), att)	0.886	0.114	−3063.8	−3084.2	20.5	297	263	-
((att, mus), dai)*	0.964	0.036	−4467.5	−4472.2	4.7	462	445	-
**((dai**,** att)**,** mus)**	**0.996**	**0.004**	**−2821.0**	**−2763.2**	**−57.8**	**266**	**265**	**1**

* The topology matches the species-tree topology

### Phylogenetic signal

According to the analysis of the phylogenetic signal, only the stigma width (SW) showed a significant Blomberg et al. (2003) *K* value greater than 1, indicating that this trait evolved with a closer adherence to the phylogeny, that is, the trait is primarily driven by evolutionary constraints or shared ancestry, and tends to follow the phylogenetic tree closely (i.e., phylogenetic conservatism, Table 2; Fig. 2b). The other traits did not show significant estimates deviating from 1 (*P* > 0.05), suggesting that they evolved following the Brownian motion model and were under genetic drift or neutral evolution.

**Table 2:** Phylogenetic signals of quantitative traits estimated by Blomberg et al. (2003) *K* with 10,000 randomization. All trait measurements were adopted from Lu et al. (2023)

Abbreviation	Trait	Blomberg et al.’s K	*P*
Leaf			
LL	Leaf length	0.483	0.827
BL/BWL	The ratio of blade length to the length from the blade base to the widest part of the blade	0.484	0.868
BL/BW	The ratio of blade length to the widest part of the blade	0.353	0.932
LL/BL	The ratio of leaf length to blade length	0.853	0.296
LL/PL	The ratio of leaf length to petiole length	0.638	0.463
Flower			
LT	Lobe thickness	1.068	0.168
SCL	The curve length of the stigma surface	0.415	0.926
LBST	The distance from the lobe base to the stigma apex	0.525	0.692
SDA	Stigma angle	0.631	0.466
SL	Stigma height	0.440	0.796
**SW**	**Stigma width**	**1.774**	0.032*
LBW	Lobe base width	0.572	0.613
PTL2/PTBW	The ratio of the perianth tube length to the width of the perianth tube base	0.830	0.218
PTW1/PTBW	The ratio of the perianth tube width on the stamen-attached position to the width of the perianth tube base	0.999	0.301
PTW2/PTBW	The ratio of the perianth tube width on the stigma apex to the width of the perianth tube base	0.655	0.508
PTW3/PTBW	The ratio of the perianth tube width on the lobe base to the width of the perianth tube base	0.702	0.338
PTL1/PH	The ratio of the perianth tube length (exclude lobe) to pistil height	0.602	0.559
PTL1/SH	The ratio of the perianth tube length (exclude lobe) to the height of the stamen attached to the perianth tube (stamen height)	0.606	0.555
PTL1/LBL	The ratio of the perianth tube length (exclude lobe) to lobe length	0.881	0.283
PTL1/PTL2	The ratio of the perianth tube length (exclude lobe) to the perianth tube length	0.484	0.814
PH/SH	The ratio of pistil height to the height of the stamen attached to the perianth tube (stamen height)	0.448	0.879

### Network analysis

The NeighborNet analysis clearly demonstrates species differentiation, with only a few misidentified samples, such as between *lgk* and *att* (Fig. 4a), despite confirmed morphology. Moreover, *lcn* was appropriately grouped with *mus*, supporting its taxonomic status as an *A. mushaensis* variety. The intricate reticulate structures in the network strongly imply interbreeding or introgression events between these species.

### Principal component analysis

According to the PCA, *att*, *lgk*, *dai*, and *mus* were separated, with *lcn* being clustered with *mus* (Fig. 4b), confirming that *mus* and *lcn* are of the same species. The first axis of PCA, which accounts for 32.5% of genetic variations, failed to distinguish *dai* and *mus*, while *lgk* is a subset of *att*. This finding aligns with NeighborNet, which clustered *lcn* with *mus* and placed *dai* closer to *mus*. These results demonstrate a genetic similarity between *dai* and *mus*.

### Bayesian clustering analysis revealed by STRUCTURE

**Fig. 5:**
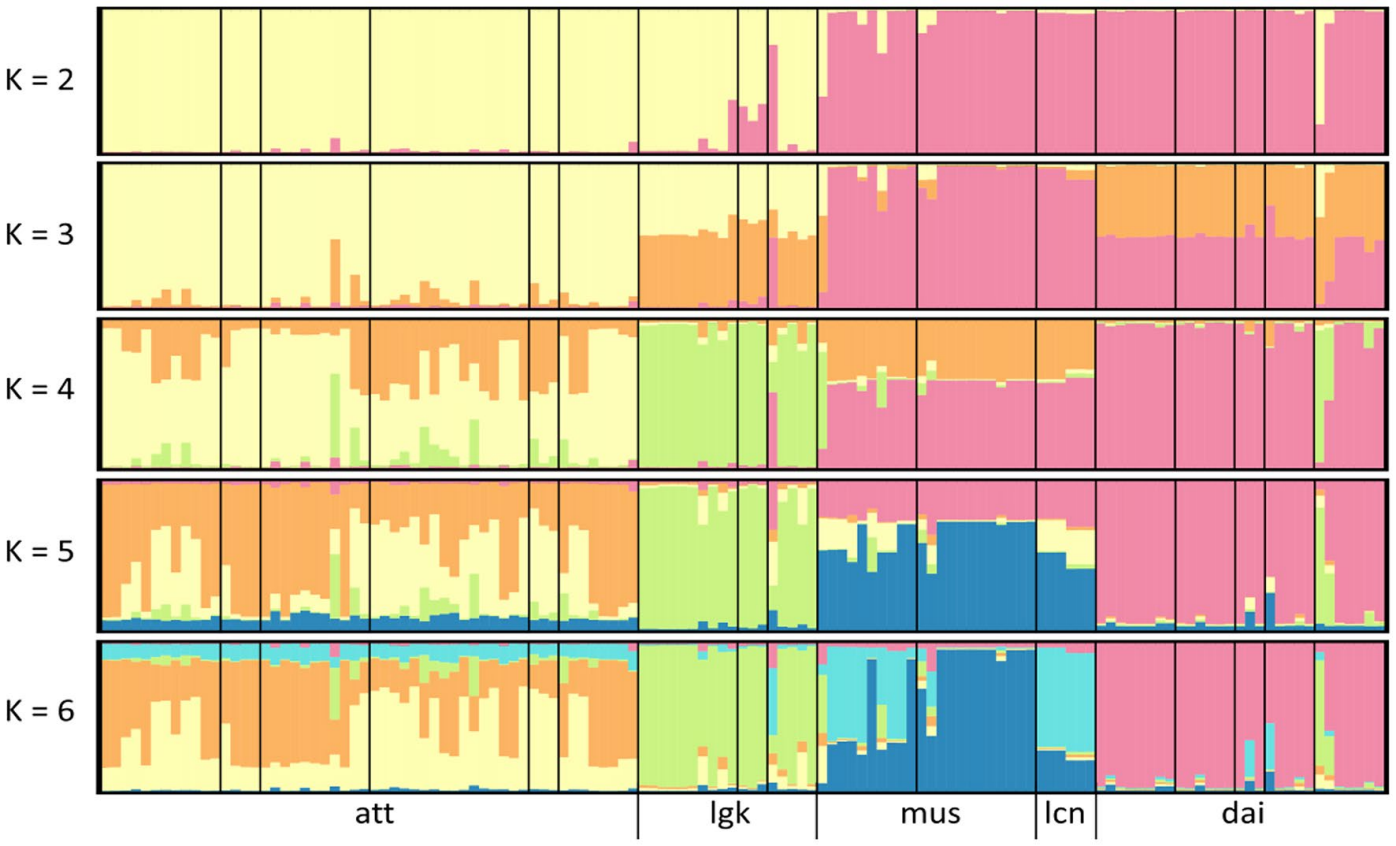
Hierarchical clustering of the STRUCTURE from *K* = 2 to *K* = 6. Data were collected from Lu et al. (2023). Abbreviations *att*, *dai*, *lgk*, *mus*, and *lcn* denote *A. attenuata*, *A. daibuensis* var. *daibuensis*, *A. daibuensis* var. *longkiauensis*, *A. mushaensis* var. *mushaensis*, and *A. mushaensis* var. *longiconnectiva*, respectively

We analyzed the SSR data of Lu et al. (2023) in different cluster numbers, ranging from *K* = 2 to *K* = 6 (Fig. 5). Our findings show that *att* and *lgk* belong to the same cluster, while *dai*, *mus*, and *lcn* belong to a different cluster when *K* = 2. However, when *K* = 3, *lgk* and *dai* showed mixed genetic components with a part of *att* and *mus* + *lcn* components, respectively. When *K* = 4, as Lu et al. (2023) interpreted, *mus* and *lcn* comprised genetic components from *dai* and *att*, which led to the hypothesis of hybridization (hypothesis 1 in ABC). However, when *K* = 5, the genetic components in *att* that contributed to *mus* became relatively small, and when *K* = 6, the genetic contribution of *dai* into *mus* was small. Our analysis of the genetic contributions at different hierarchical clustering levels suggests that the genetic mixing may not solely be attributed to hybridization but could be more complicated by ancestral polymorphism or ILS.

### Approximate bayesian computation

**Fig. 6:**
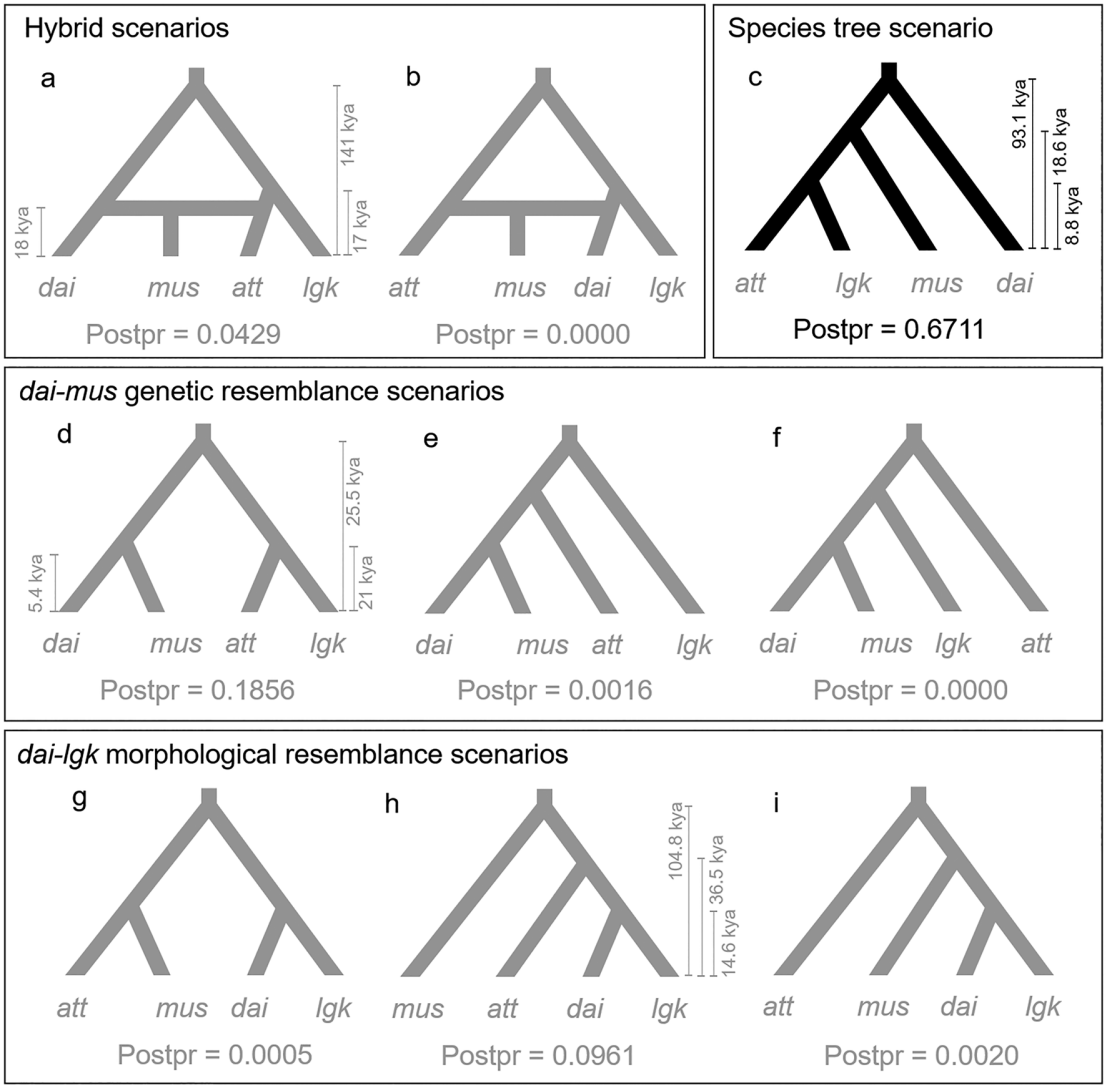
Nine scenarios of the evolutionary relationship of *Aspidistra* in Taiwan. For simplify the model, *A. mushaensis* var. *longiconnectiva* (*lcn*) was incoporated into *A. mushaensis* (*mus*). These nine scenarios represent four evolutionary hypotheses: H1: hybrid scenarios (**a** and **b**); H2: species tree scenario (**c**); H3: *dai-mus* genetic resemblance scenarios (**d**, **e**, and **f**); H4: *dai-lgk* morphological resemblance scenarios (**g**, **h**, and **i**). The divergence time estimations were conducted for the best scenario of each hypothesis. The best scenario of the nine scenarios was coloured black, while the others were grey. Abbreviations *att*, *dai*, *lgk*, and *mus* denote *A. attenuata*, *A. daibuensis* var. *daibuensis*, *A. daibuensis* var. *longkiauensis*, and *A. mushaensis*, respectively

The ABC suggests scenario **c** as the most probable (posterior probability 0.6711) (Fig. 6). Parameter estimation of the best model shows *att* diverged from *lgk* 8.8 kya, *mus* 18.6 kya, and *dai* 93.1 kya, given a generation time of one year (Fig. 6c). Scenario **d** of the *dai-mus* genetic resemblance scenarios scored the second-highest probability of 0.1856 (Fig. 6d). The top two scenarios cluster *lgk* with *att* instead of with *dai* that reject the morphological resemblance scenarios between *dai* and *lgk*, which classify them as varieties of *A. daibuensis*.

### Taxonomic treatment

***Aspidistra longkiauensis*** (C.T. Lu, Ming Jen Yang & J.C. Wang) C.T. Lu & J.C. Wang, *stat. nov*.

Basionym *Aspidistra daibuensis* Hayata var. *longkiauensis* C.T.Lu, Ming Jen Yang & J.C.Wang, PhytoKeys 222: 129–151. 2023.

**Type material examined** Taiwan. Pintung County, Shuangliou National Forest Recreation Area, Banyan Trail, elev. 200–300 m, 12 Jun 2020, *M.J.Yang s.n.* (holotype: TAIF; isotype: TNU).

#### Distribution and habitat

*Aspidistra longkiauensis* is native to the Hengchun Peninsula and southeastern Taiwan. It grows on forested slopes at elevations of 200 to 500 m.

#### Note

This species resembles *A. daibuensis*, but it has a shorter leaf length (72.24 ± 11.68 cm vs. 95.46 ± 21.04 cm), a smaller leaf blade length-to-width ratio (3.73 ± 1.43 vs. 8.05 ± 1.73), thinner perianth lobes (1.28 ± 0.38 mm vs. 2.79 ± 0.64 mm), and a longer stigma curvature (10.98 ± 1.38 mm vs. 16.23 ± 1.45 mm) and narrower stigma width (10.33 ± 1.79 mm vs. 16.43 ± 2.21 mm). It also has a larger pistil-to-stamen height ratio (2.49 ± 0.59 vs. 2.03 ± 0.78). Additionally, it is also similar to *A. mushaensis*, but this species differs in its smaller leaf blade ratio (3.73 ± 1.43 vs. 7.66 ± 1.35), narrower perianth lobe base (5.12 ± 1.12 mm vs. 7.27 ± 1.19 mm), and larger pistil-to-stamen height ratio (2.49 ± 0.59 vs. 1.59 ± 0.26).

## Discussion

### Phylogenetic inference by gene genealogy interrogation (GGI)

While the gene tree topology, which comprises 189 genes, is consistent with the species tree derived from the MSC method inferred by ASTRAL, more than 80% of the genes support alternative tree topologies in the AU test. As the GGI strategy, the AU test ranks alternative resolutions supported by individual genes, revealing support distribution for different topologies and mitigating gene tree estimation errors in coalescent analyses (Betancur-R et al. 2019). Thus, only 189 genes were utilized to determine the divergence times among species through dating with the MSC tree. This revealed very recent divergent times among species (Bagley et al. 2020). On the other hand, the genes that did not conform to the MSC species tree topology indicate a notable occurrence of ILS in these closely related species (Degnan and Salter 2005). However, the proportion of ILS in this case (> 90%, Table 1) is much higher than the estimation of approximately 10% by Rosenfeld et al. (2012).

The AU test inferred that the top three supported topologies clustered *mus*, *att*, and *lgk*, with a distant phylogenetic taxon, *dai*, backed by 189, 150, and 119 genes. It is noteworthy that these three species (*mus*, *att*, and *lgk*) are predominantly located on the western side of Taiwan’s Central Mountain Range, compared to the east-distributed *dai*, indicating that these 458 genes could potentially distinguish the diverse environments between the eastern and western Taiwan, although the common characteristics of these genes are yet to be determined. In addition, the ABC result also suggests that *mus*, *att*, and *lgk* had a very recent coalescence time (18.6 kya) during the LGM period, in contrast to the relatively long divergence of *dai* (93.1 kya) (Fig. 6c). Even in the BEAST analysis, the divergence among *mus*, *att*, and *lgk* was 154.3 kya, much shorter than the divergence time of 281.3 kya from *dai* (Fig. 2a).

It is believed that the eastern-distributed *dai* was the first to diverge from the other species. As the diversity center of *Aspidistra* was located in southern regions like Guangxi Province of China and Vietnam, the ancestral area of *mus*, *lcn*, *att*, and *lgk* was likely in the south of Taiwan. Moreover, since the Center Mountain Range of Taiwan acts as a barrier, it is less feasible for the species to disperse westward across the mountains. Therefore, based on the tree topology, after *mus* and *lcn* branched off from *att* and *lgk*, they may have expanded northward in western Taiwan. The current distribution trends are a direct result of the postglacial dispersion of the common ancestor, with the environment potentially playing a significant role.

It is important to note that only stigma width demonstrates a strong correlation with species relationships among the morphological traits considered. While other features may differentiate species, they are all subject to random variation under the Brownian motion model. Speciation is the natural ecological process that leads to biodiversity synthesis (Swenson 2011), and traits that have evolved alongside this process, such as the stigma width of Taiwanese *Aspidistra*, may be more suitable as diagnostic characters for taxonomy (e.g., Zhang et al. 2016). In traditional taxonomical studies, the shape of the stigma has been used as a critical character for the species-level classification of *Aspidistra*. This study supports this view.

The width of the stigma plays a crucial role in filtering the body size of pollinators since the anthers of *Aspidistra* are below the stigma. The evolution of stigma width could be critical for the reproductive barriers between *Aspidistra* species. Importantly, the conservative nature of stigma width suggests that this trait may provide a practical and reliable morphological marker in future taxonomic revisions of *Aspidistra*. When combined with broader geographic and genomic sampling, stigma width has the potential to facilitate integrative approaches to resolving species boundaries across the genus. On the other hand, the other morphological traits can exhibit plasticity during evolution in response to environmental changes. Such changes are not necessarily evolutionarily constrained and can lead to homoplasious characters (Brandley et al. 2009). Although these traits may help distinguish a single species, they are unsuitable for identifying an entire species group.

### Raising the taxonomic rank of *A. daibuensis* var. *longkiauensis* to *A. longkiauensis*

Genetic evidence suggests that *dai* and *lgk* may not be considered a single but two potentially independent species. However, because of the morphological resemblance, *dai* (*A. daibuensis* var. *daibuensis*) and *lgk* (var. *longkiauensis*) were considered as varieties of the same species previously (i.e., *dai-lgk* morphological resemblance hypothesis). This distinction could be attributed to similarities in certain genes, leading to homoplasious characteristics within a phylogenetic context (Wake et al. 2011). In our study system, the SSR dataset partially supports the hypothesis that *dai* and *lgk* share morphological similarities, with an approximate 10% posterior probability in ABC analysis. On the other hand, the RNAseq dataset indicates that 20.8% of genes do not reject the idea of clustering *dai* and *lgk* together in their phylogeny. Among these genes, we have identified multiple genes that play a role in chloroplastic functions, suggesting a convergence in photosynthetic processes, such as electron transfer (*AOX4* gene) and redox reactions (*ACHT2* gene), as well as chlorophyll-related processes, including chlorophyll degradation (*PAO* gene), in both taxa. Additionally, the nuclear gene *DET1*, responsible for encoding a light-mediated development protein involved in fruit pigmentation and nutritional quality, also appears to be linked to morphogenesis. Specifically, four other genes, *ELIP1*, *Ij*, *PBAC5*, and *PRMT11*, were detected to be positively selected. ELIP1 plays a crucial role in the cellular response to light and regulates chlorophyll biosynthesis (Casazza et al. 2005) and seed germination (Rizza et al. 2011). Iojap is a chloroplastic gene known for its unique green and white striped leaf pattern (Han et al. 1992). PBAC5 is a period circadian protein highly expressed in the floral, leaves, and seed parts (Klepikova et al. 2016) and is involved in the function of the chaperone (Gemperline et al. 2019). These three genes share a light response trait, potentially impacting the plant’s coloration. *PRMT11* functions in epigenetics (DNA methylation) and arginine methylation (Scebba et al. 2007). The *Aspidistra* plant thrives in shaded areas of forest floors and is not well-suited to direct sunlight. Changes in amino acids on certain genes indicate adaptive signals for the *dai* and *lgk* branches. Although the exact link between these genes and the physical similarities of *dai* and *lgk* is still not fully comprehended, their resemblance may be related to their physiological and photomorphogenic co-adaptation.

Despite morphological similarity, the ABC analysis supports a non-monophyletic relationship between *A. daibuensis* var. *daibuensis* and var. *longkiauensis*. The result matches the MSC species tree from 1,029 coding sequences, although only 18.4% of genes confirmed the species tree topology in the AU test. It is possible that the ILS and ancient interbreeding could be the reason for the high percentage of genetic inconsistency with the species tree topology (Edwards et al. 2007). A relatively short divergence time (< 1 Ma) implied that multiple genes may not have had enough time to differentiate completely, leading to ILS (Bagley et al. 2020; e.g., Murillo-A et al. 2022). This, in turn, means no substantial genetic barriers preventing gene flow between species, which can cause genetic leaks across reproductive barriers (Wu and Ting 2004).

However, the hybrid scenario inferred by STRUCTURE was rejected in ABC with a relatively low posterior probability. Lu et al. (2023) had shown that *mus* was comprised of genetic components from *att* and *dai*. Thus, a hybrid hypothesis could have arisen. However, only 4% posterior probability supports this hypothesis in ABC. QuIBL also suggests a high frequency of ILS rather than introgression, indicating that the genetic leaks across the reproductive barrier only occurred between gene levels instead of species levels. Thus, the frequent hybridization may not explain the reticulate evolution revealed in the NeighborNet. It may result from the ILS due to the short divergence time. In fact, the interbreeding between species was also less observed in the field. Evident geographical isolation confined small-area distribution may accelerate species differentiation, which may be explained by genetic drift of small populations and local adaptation (e.g., Liu et al. 2022), but further research is needed.

## Conclusion

In conclusion, our study provides valuable insights into the complex evolutionary dynamics and taxonomic relationships within the Taiwanese *Aspidistra* species group. Phylogenetic inference through GGI revealed that while the gene tree topology generally aligns with the species tree derived from the MSC method, a substantial proportion of genes supported alternative tree topologies, suggesting the presence of ILS in these closely related species. The genetic evidence suggests that *A. daibuensis* var. *daibuensis* and var. *longkiauensis* should not be considered a single species, as previously believed, due to morphological resemblance. Instead, they exhibit non-monophyletic relationships.

Interestingly, certain genes related to chloroplastic functions and photomorphogenic co-adaptation were identified, shedding light on potential physiological and adaptive similarities between these taxa. Additionally, our findings emphasize the importance of stigma shape, particularly stigma width, as a diagnostic character in traditional species-level taxonomy. This trait is also recognized as phylogenetically conservative. It may play a crucial role in future integrative taxonomic revisions, especially when combined with more extensive sampling across Asia. This characteristic might offer stronger indications of speciation events compared to other morphological features subject to random variation.

Notably, our phylogenomic analyses indicate that *A. longkiauensis* is distinct from *A. daibuensis* within the Taiwanese clade. However, our findings are based solely on endemic species from Taiwan. Given that *Aspidistra* includes over 200 species across Asia, our conclusions are provisional and require broader pan-Asian sampling. Expanding taxonomic and geographic coverage will be crucial to assess whether the evolutionary patterns observed are consistent across the genus and to improve the systematic framework of *Aspidistra*. Overall, this study highlights the intricate interplay of genetics, morphology, and ecology in the evolution of closely related plant species and calls for further research to elucidate the underlying mechanisms driving their diversification and adaptation.

## Supplementary Information

Below is the link to the electronic supplementary material.

Supplementary Material 1: **Table S1** Sampling and sequencing information. **Table S2** Annotation of 214 genes that did not reject the monophyletic relationship between *dai* and *lgk*.

Supplementary Material 2: **Figure S1** Convergence plot for two independent runs in MCMCTree. **Figure S2** Prior–posterior plots for node age mean distributions of MCMCTree. **Figure S3** Species tree reconstructed by ASTRAL with 1,029 genes.

## Data Availability

Raw sequence files can be accessed from the NCBI Sequence Read Archive under the BioProject accession number PRJNA1066607.

## Abbreviations

ABC: Approximate Bayesian Computation
*ACHT2*: *Atypical Cys His-rich Trx-2* (*Thioredoxin-like 2–2*)
*AOX4*: *Ubiquinol oxidase 4*
*att*: *Aspidistra attenuata*
AU: Approximately unbiased
BIC: Bayesian information criterion
CI: Confidence interval
*dai*: *Aspidistra daibuensis* var. *daibuensis*
*DET1*: *DEETIOLATED 1*
*ELIP1*: *EARLY LIGHT-INDUCIBLE PROTEIN*
ESS: Effective sample size
GGI: Gene genealogy interrogation
*Ij*: *IOJAP*
ILS: Incomplete lineage sorting
*K*: The number of subpopulations
*Ka/Ks*: The relative rates of synonymous and nonsynonymous substitutions at a particular site, an index of positive selection
kya: Kilo-years ago
*lcn*: *Aspidistra mushaensis* var. *longiconnectiva*
*lgk*: *Aspidistra daibuensis* var. *longkiauensis*
MCMC: Markov Chain Monte Carlo
MRCA: The most recent common ancestor
MSC: Multispecies coalescent
*mus*: *Aspidistra mushaensis* var. *mushaensis*
Ma: Mega-annum
OTU: Operational taxonomic unit
*PAO*: *PHEOPHORBIDE A OXYGENASE*
*PBAC5*: *PROTEASOME BIOGENESIS-ASSOCIATED CHAPERONE 5*
PCA: Principal component analysis
*PRMT11*: *PROTEIN ARGININE N-METHYLTRANSFERASE 1.1*
RELL: Resamplings of estimated log-likelihoods
SSR: Simple-sequence repeats
SW: Stigma width
TAIR: The *Arabidopsis* Information Resource
*μ*: The average rate of silent-site divergence

## References

[CR1] Andrews S (2010) FastQC: a quality control tool for high throughput sequence data. Babraham Bioinformatics, Babraham Institute, Cambridge, United Kingdom

[CR2] Arcila D, Ortí G, Vari R, Armbruster JW, Stiassny MLJ, Ko KD, Sabaj MH, Lundberg J, Revell LJ, Betancur-R, R (2017) Genome-wide interrogation advances resolution of recalcitrant groups in the tree of life. Nat Ecol Evol 1:0020 10.1038/s41559-016-0020 28812610

[CR3] Avise JC, TJ Robinson (2008) Hemiplasy: A new term in the lexicon of phylogenetics. Syst Biol 57:503–507 18570042 10.1080/10635150802164587

[CR4] Bagley JC, Uribe-Convers S, Carlsen MM, Muchhala N (2020) Utility of targeted sequence capture for phylogenomics in rapid, recent angiosperm radiations: Neotropical burmeistera bellflowers as a case study. Mol Phylogenet Evol 152:106769 32081762 10.1016/j.ympev.2020.106769

[CR5] Berardini TZ, Reiser L, Li D, Mezheritsky Y, Muller R, Strait E, Huala E (2015) The Arabidopsis information resource: making and mining the gold standard annotated reference plant genome. Genesis 53:474–485 26201819 10.1002/dvg.22877 PMC4545719

[CR6] Betancur -RR, Arcila D, Vari RP, Hughes LC, Oliveira C, Sabaj MH, G Ortí (2019) Phylogenomic incongruence, hypothesis testing, and taxonomic sampling: the monophyly of characiform fishes*. Evolution 73:329–345 30426469 10.1111/evo.13649

[CR7] Blomberg SP, Garland Jr T, AR Ives (2003) Testing for phylogenetic signal in comparative data: behavioral traits are more labile. Evolution 57:717–745 12778543 10.1111/j.0014-3820.2003.tb00285.x

[CR9] Bolger AM, Lohse M, Usadel B (2014) Trimmomatic: a flexible trimmer for illumina sequence data. Bioinformatics 30:2114–2120 24695404 10.1093/bioinformatics/btu170 PMC4103590

[CR11] Bouckaert RR (2010) DensiTree: making sense of sets of phylogenetic trees. Bioinformatics 26:1372–1373 20228129 10.1093/bioinformatics/btq110

[CR10] Bouckaert R, Vaughan TG, Barido-Sottani J, Duchêne S, Fourment M, Gavryushkina A, Heled J, Jones G, Kühnert D, N De Maio (2019) BEAST 2.5: an advanced software platform for bayesian evolutionary analysis. PLoS Comput Biol 15:e1006650 30958812 10.1371/journal.pcbi.1006650 PMC6472827

[CR12] Brandley MC, Warren DL, Leaché AD, JA McGuire (2009) Homoplasy and clade support. Syst Biol 58:184–198 20525577 10.1093/sysbio/syp019

[CR13] Camacho C, Coulouris G, Avagyan V, Ma N, Papadopoulos J, Bealer K, TL Madden (2009) BLAST+: architecture and applications. BMC Bioinformatics 10:1–9 20003500 10.1186/1471-2105-10-421 PMC2803857

[CR14] Casazza AP, Rossini S, Rosso MG, Soave C (2005) Mutational and expression analysis of ELIP1 and ELIP2 in *Arabidopsis thaliana*. Plant Mol Biol 58:41–51 16028115 10.1007/s11103-005-4090-1

[CR15] Csilléry K, François O, Blum MG (2012) Abc: an R package for approximate bayesian computation (ABC). Methods Ecol Evol 3:475–479 10.1016/j.tree.2010.04.001 20488578

[CR16] Darriba D, Taboada G, Doallo R, Posada D (2012) jModelTest 2: more models, new heuristics and parallel computing. Nat Methods 9(8):772 22847109 10.1038/nmeth.2109 PMC4594756

[CR17] De La Torre AR, Li Z, Van de Y, Peer PK, Ingvarsson (2017) Contrasting rates of molecular evolution and patterns of selection among gymnosperms and flowering plants. Mol Biol Evol 34:1363–1377 28333233 10.1093/molbev/msx069 PMC5435085

[CR18] Degnan JH, Salter LA (2005) Gene tree distributions under the coalescent process. Evolution 59:24–37 15792224

[CR19] Doroodian P, Z Hua (2021) The ubiquitin switch in plant stress response. Plants 246:10 10.3390/plants10020246 PMC7911189 33514032

[CR20] Douglas J, Jiménez-Silva CL, Bouckaert R (2022) StarBeast3: adaptive parallelized bayesian inference under the multispecies coalescent. Syst Biol 71:901–916 35176772 10.1093/sysbio/syac010 PMC9248896

[CR21] Durand EY, Patterson N, Reich D, Slatkin M (2011) Testing for ancient admixture between closely related populations. Mol Biol Evol 28:2239–2252 21325092 10.1093/molbev/msr048 PMC3144383

[CR22] Edelman NB, Frandsen PB, Miyagi M, Clavijo B, Davey J, Dikow RB, García-Accinelli G, Van Belleghem SM, Patterson N, Neafsey DE, Challis R, Kumar S, Moreira GRP, Salazar C, Chouteau M, Counterman BA, Papa R, Blaxter M, Reed RD, Dasmahapatra KK, Kronforst M, Joron M, Jiggins CD, McMillan WO, Palma FD, Blumberg AJ, Wakeley J, Jaffe D, J Mallet (2019) Genomic architecture and introgression shape a butterfly radiation. Science 366:594–599 31672890 10.1126/science.aaw2090 PMC7197882

[CR23] Edwards SV, Liu L, Pearl DK (2007) High-resolution species trees without concatenation. Proc Natl Acad Sci 104:5936–5941 10.1073/pnas.0607004104 PMC1851595 17392434

[CR24] Emms DM, Kelly S (2019) OrthoFinder: phylogenetic orthology inference for comparative genomics. Genome Biol 20:1–14 31727128 10.1186/s13059-019-1832-y PMC6857279

[CR25] Ewels P, Magnusson M, Lundin S, M Käller (2016) MultiQC: summarize analysis results for multiple tools and samples in a single report. Bioinformatics 32:3047–3048 27312411 10.1093/bioinformatics/btw354 PMC5039924

[CR27] Excoffier L, M Foll (2011) Fastsimcoal: a continuous-time coalescent simulator of genomic diversity under arbitrarily complex evolutionary scenarios. Bioinformatics 27:1332–1334 21398675 10.1093/bioinformatics/btr124

[CR26] Excoffier L, Lischer HE (2010) Arlequin suite ver 3.5: a new series of programs to perform population genetics analyses under Linux and windows. Mol Ecol Resour 10:564–567 21565059 10.1111/j.1755-0998.2010.02847.x

[CR28] Excoffier L, Dupanloup I, Huerta-Sánchez E, Sousa VC, Foll M (2013) Robust demographic inference from genomic and SNP data. PLoS Genet 9:e1003905 24204310 10.1371/journal.pgen.1003905 PMC3812088

[CR29] Fu L, Niu B, Zhu Z, Wu S, Li W (2012) CD-HIT: accelerated for clustering the next-generation sequencing data. Bioinformatics 28:3150–3152 23060610 10.1093/bioinformatics/bts565 PMC3516142

[CR30] Gemperline DC, Marshall RS, Lee K-H, Zhao Q, Hu W, McLoughlin F, Scalf M, Smith LM, Vierstra RD (2019) Proteomic analysis of affinity-purified 26S proteasomes identifies a suite of assembly chaperones in *Arabidopsis*. J Biol Chem 294:17570–17592 31562246 10.1074/jbc.RA119.010219 PMC6873196

[CR31] Grabherr MG, Haas BJ, Yassour M, Levin JZ, Thompson DA, Amit I, Adiconis X, Fan L, Raychowdhury R, Q Zeng (2011) Full-length transcriptome assembly from RNA-Seq data without a reference genome. Nat Biotechnol 29:644–652 21572440 10.1038/nbt.1883 PMC3571712

[CR32] Guindon S, Gascuel O (2003) A simple, fast, and accurate algorithm to estimate large phylogenies by maximum likelihood. Syst Biol 52:696–704 14530136 10.1080/10635150390235520

[CR33] Haas BJ (2013) TransDecoder. http://transdecoder.sf.net

[CR34] Hahn MW, MS Hibbins (2019) A Three-Sample test for introgression. Mol Biol Evol 36:2878–2882 31373630 10.1093/molbev/msz178

[CR35] Han CD, Coe Eh RA Jr, Fau - Martienssen RA, Martienssen (1992) Molecular cloning and characterization of *Iojap* (*ij*), a pattern striping gene of maize. EMBO J 11:4037–4046 1382980 10.1002/j.1460-2075.1992.tb05497.x PMC556914

[CR36] Harmon L, Weir J, Brock C, Glor R, Challenger W, Hunt G, FitzJohn R, Pennell M, Slater G, Brown J (2009) geiger: Analysis of evolutionary diversification. R package version 1

[CR37] Hibbins MS, Hahn MW (2022) Phylogenomic approaches to detecting and characterizing introgression. Genetics 220:iyab173 34788444 10.1093/genetics/iyab173 PMC9208645

[CR38] Huang D, Song Z, Wang Y, Chen J (2013) Phylogenetic analysis of *Aspidistra* inferred from sequences of RLCKVII gene. J Fudan Univ (Natural Science) 52:436–441

[CR39] Huson DH, Bryant D (2006) Application of phylogenetic networks in evolutionary studies. Mol Biol Evol 23:254–267 16221896 10.1093/molbev/msj030

[CR40] Ji Y, Landis JB, Yang J, Wang S, Zhou N, Luo Y, Liu H (2022) Phylogeny and evolution of Asparagaceae subfamily nolinoideae: new insights from plastid phylogenomics. Ann Botany 131:301–312 10.1093/aob/mcac144 PMC9992941 36434782

[CR41] Jombart T (2008) Adegenet: a R package for the multivariate analysis of genetic markers. Bioinformatics 24:1403–1405 18397895 10.1093/bioinformatics/btn129

[CR42] Kalyuzhny S, Vislobokov N, Luu HT, Plugatar Y, Kuznetsov A, Kuznetsova S, Korzhenevsky V, Vin’Kovskaya (2022) *Aspidistra nikitensis* (Asparagaceae, Nolinoideae), a new species from Vietnam. Phytotaxa 574:289–294

[CR43] Kishino H, Miyata T, Hasegawa M (1990) Maximum likelihood inference of protein phylogeny and the origin of chloroplasts. J Mol Evol 31:151–160

[CR44] Klepikova AV, Kasianov AS, Gerasimov ES, Logacheva MD, Penin AA (2016) A high resolution map of the *Arabidopsis thaliana* developmental transcriptome based on RNA-seq profiling. Plant J 88:1058–1070 27549386 10.1111/tpj.13312

[CR45] Li G (2004) The genus *Aspidistra*. Guangxi Science & Technology Publishing House, Nanning, China, p 229

[CR47] Li W, Godzik A (2006) Cd-hit: a fast program for clustering and comparing large sets of protein or nucleotide sequences. Bioinformatics 22:1658–1659 16731699 10.1093/bioinformatics/btl158

[CR46] Li N, Li Y (2014) Ubiquitin-mediated control of seed size in plants. Front Plant Sci 5:332 25071811 10.3389/fpls.2014.00332 PMC4093792

[CR48] Liu X, Zhang S, Cai Z, Kuang Z, Wan N, Wang Y, Mao L, An X, Li F, Feng T, Liang X, Qiao Z, Nevo E, Li K (2022) Genomic insights into zokors’ phylogeny and speciation in China. Proc Natl Acad Sci U S A 119:e2121819119 10.1073/pnas.2121819119 PMC9171634 35512099

[CR50] Lu C-T, Chuang K-C, Tseng Y-H, Wang C-C, Wang J-C (2020) Taxonomic revision of *Aspidistra* Ker-Gawl. (Asparagaceae) in Taiwan. Taiwania 65:277–285

[CR49] Lu C-T, Yang M-J, Luo M-X, Wang J-C (2023) *Aspidistra daibuensis* var. *longkiauensis*, a new variety of *Aspidistra* (Asparagaceae) from Taiwan, identified through morphological and genetic analyses. PhytoKeys 222:129–151 37215050 10.3897/phytokeys.222.100885 PMC10194778

[CR51] Maddison WP, LL Knowles (2006) Inferring phylogeny despite incomplete lineage sorting. Syst Biol 55:21–30 16507521 10.1080/10635150500354928

[CR52] Minh BQ, Schmidt HA, Chernomor O, Schrempf D, Woodhams MD, Von Haeseler A, Lanfear R (2020) IQ-TREE 2: new models and efficient methods for phylogenetic inference in the genomic era. Mol Biol Evol 37:1530–1534 32011700 10.1093/molbev/msaa015 PMC7182206

[CR53] Mirarab S, Warnow T (2015) ASTRAL-II: coalescent-based species tree estimation with many hundreds of taxa and thousands of genes. Bioinformatics 31:i44–i52 26072508 10.1093/bioinformatics/btv234 PMC4765870

[CR54] Murillo -AJ, Valencia-D J, Orozco CI, Parra-O C, Neubig KM (2022) Incomplete lineage sorting and reticulate evolution mask species relationships in Brunelliaceae, an Andean family with rapid, recent diversification. Am J Bot 109:1139–1156 35709353 10.1002/ajb2.16025

[CR55] Nguyen L-T, Schmidt HA, Haeseler AV, Minh BQ (2015) IQ-TREE: a fast and effective stochastic algorithm for estimating maximum-likelihood phylogenies. Mol Biol Evol 32:268–274 25371430 10.1093/molbev/msu300 PMC4271533

[CR56] Pagès H, Aboyoun P, Gentleman R, DebRoy S (2025) Biostrings: Efficient manipulation of biological strings. R package version 2.76.0. https://bioconductor.org/packages/Biostrings

[CR57] Paradis E, Schliep K (2019) Ape 5.0: an environment for modern phylogenetics and evolutionary analyses in R. Bioinformatics 35:526–528 30016406 10.1093/bioinformatics/bty633

[CR58] Peakall R, Smouse PE (2006) GENALEX 6: genetic analysis in Excel. Population genetic software for teaching and research. Mol Ecol Notes 6:288–295 10.1093/bioinformatics/bts460 PMC3463245 22820204

[CR59] Pease JB, Hahn MW (2015) Detection and polarization of introgression in a five-taxon phylogeny. Syst Biol 64:651–662 25888025 10.1093/sysbio/syv023

[CR60] Philippe H, Brinkmann H, Lavrov DV, Littlewood DTJ, Manuel M, Wörheide G, Baurain D (2011) Resolving difficult phylogenetic questions: why more sequences are not enough. PLoS Biol 9:e1000602 21423652 10.1371/journal.pbio.1000602 PMC3057953

[CR61] Pokhilko A, Ramos JA, Holtan H, Maszle DR, Khanna R, AJ Millar (2011) Ubiquitin ligase switch in plant photomorphogenesis: A hypothesis. J Theor Biol 270:31–41 21093457 10.1016/j.jtbi.2010.11.021 PMC3021735

[CR62] Rambaut A, Drummond AJ, Xie D, Baele G, Suchard MA (2018) Posterior summarization in bayesian phylogenetics using tracer 1.7. Syst Biol 67:901–904 29718447 10.1093/sysbio/syy032 PMC6101584

[CR63] Rannala B, Yang Z (2007) Inferring speciation times under an episodic molecular clock. Syst Biol 56:453–466 17558967 10.1080/10635150701420643

[CR64] Ranwez V, Harispe S, Delsuc F, Douzery EJ (2011) MACSE: multiple alignment of coding sequences accounting for frameshifts and stop codons. PLoS ONE 6:e22594 21949676 10.1371/journal.pone.0022594 PMC3174933

[CR65] Reddy S, Kimball RT, Pandey A, Hosner PA, Braun MJ, Hackett SJ, Han K-L, Harshman J, Huddleston CJ, Kingston S, Marks BD, Miglia KJ, Moore WS, Sheldon FH, Witt CC, Yuri T, Braun EL (2017) Why do phylogenomic data sets yield conflicting trees? Data type influences the avian tree of life more than taxon sampling. Syst Biol 66:857–879 28369655 10.1093/sysbio/syx041

[CR66] Reis Md Z, Yang (2011) Approximate likelihood calculation on a phylogeny for bayesian estimation of divergence times. Mol Biol Evol 28:2161–2172 21310946 10.1093/molbev/msr045

[CR67] Rizza A, Boccaccini A, Lopez-Vidriero I, Costantino P, Vittorioso P (2011) Inactivation of the ELIP1 and ELIP2 genes affects Arabidopsis seed germination. New Phytol 190:896–905 21299564 10.1111/j.1469-8137.2010.03637.x

[CR68] Rosenfeld JA, Payne A, R DeSalle (2012) Random roots and lineage sorting. Mol Phylogenet Evol 64:12–20 22445448 10.1016/j.ympev.2012.02.029

[CR69] Sackton TB, Clark N (2019) Convergent evolution in the genomics era: new insights and directions. Philos Trans R Soc B: Biol Sci 374:20190102 10.1098/rstb.2019.0102 PMC6560275 31154976

[CR70] Salichos L, Rokas A (2013) Inferring ancient divergences requires genes with strong phylogenetic signals. Nature 497:327–331 23657258 10.1038/nature12130

[CR71] Scebba F, De Bastiani M, Bernacchia G, Andreucci A, Galli A, Pitto L (2007) PRMT11: a new Arabidopsis MBD7 protein partner with arginine methyltransferase activity. Plant J 52:210–222 17711414 10.1111/j.1365-313X.2007.03238.x

[CR73] Schliep KP (2011) Phangorn: phylogenetic analysis in R. Bioinformatics 27:592–593 21169378 10.1093/bioinformatics/btq706 PMC3035803

[CR72] Schliep K, Potts AA, Morrison DA, Grimm GW (2017) Intertwining phylogenetic trees and networks. Methods Ecol Evol 8:1212–1220

[CR74] Shimizu-Inatsugi R, Morishima A, Mourato B, Shimizu KK, Sato Y (2023) Phenotypic variation of a new synthetic allotetraploid *Arabidopsis kamchatica* enhanced in natural environment. Front Plant Sci 13:1058522 36684772 10.3389/fpls.2022.1058522 PMC9846130

[CR75] Shimodaira H (2002) An approximately unbiased test of phylogenetic tree selection. Syst Biol 51:492–508 12079646 10.1080/10635150290069913

[CR76] Steenwyk JL, Li Y, Zhou X, Shen X-X, Rokas A (2023) Incongruence in the phylogenomics era. Nat Rev Genet 24:834–850 37369847 10.1038/s41576-023-00620-x PMC11499941

[CR77] Swenson NG (2011) The role of evolutionary processes in producing biodiversity patterns, and the interrelationships between taxonomic, functional and phylogenetic biodiversity. Am J Bot 98:472–480 21613140 10.3732/ajb.1000289

[CR78] Tillich H-J (2014) The genus *Aspidistra* Ker-Gawl. (Asparagaceae) in Vietnam. Taiwania 59:1–8

[CR79] Wake DB, Wake MH, Specht CD (2011) Homoplasy: from detecting pattern to determining process and mechanism of evolution. Science 331:1032–1035 21350170 10.1126/science.1188545

[CR80] Wang F, Deng XW (2011) Plant ubiquitin-proteasome pathway and its role in Gibberellin signaling. Cell Res 21:1286–1294 21788985 10.1038/cr.2011.118 PMC3193469

[CR81] Wickham H, Chang W, Wickham MH (2016) Package ‘ggplot2’. Create Elegant Data Visualisations Using Gramm Graphics Version 2:1–189

[CR82] Wu CI, CT Ting (2004) Genes and speciation. Nat Rev Genet 5:114–122 14735122 10.1038/nrg1269

[CR83] Yu G, Smith DK, Zhu H, Guan Y, Lam TTY (2017) Ggtree: an R package for visualization and annotation of phylogenetic trees with their covariates and other associated data. Methods Ecol Evol 8:28–36

[CR84] Zhang F-P, Huang J-L, Zhang S-B (2016) Trait evolution in the slipper Orchid *P**aphiopedilum* (Orchidaceae) in China. Plant Signal Behav 11:e1149668 26855188 10.1080/15592324.2016.1149668 PMC4883940

