# Supplementary material for: Impact of incomplete lineage sorting and natural selection on the phylogenetic and taxonomic uncertainties of Aspidistra in Taiwan
Source: Bot Stud. 2025 Oct 15;66:35. doi: 10.1186/s40529-025-00482-y (PMC12528535; doi:10.1186/s40529-025-00482-y)
Supplement: Supplementary file 2 — Supplementary Material 2: Figure S1 Convergence plot for two independent runs in MCMCTree. Figure S2 Prior–posterior plots for node age mean distributions of MCMCTree. Figure S3 Species tree reconstructed by ASTRAL with 1,029 genes. [file 40529_2025_482_MOESM2_ESM.docx]

**Figure S1** Convergence plot for two independent runs in MCMCTree.


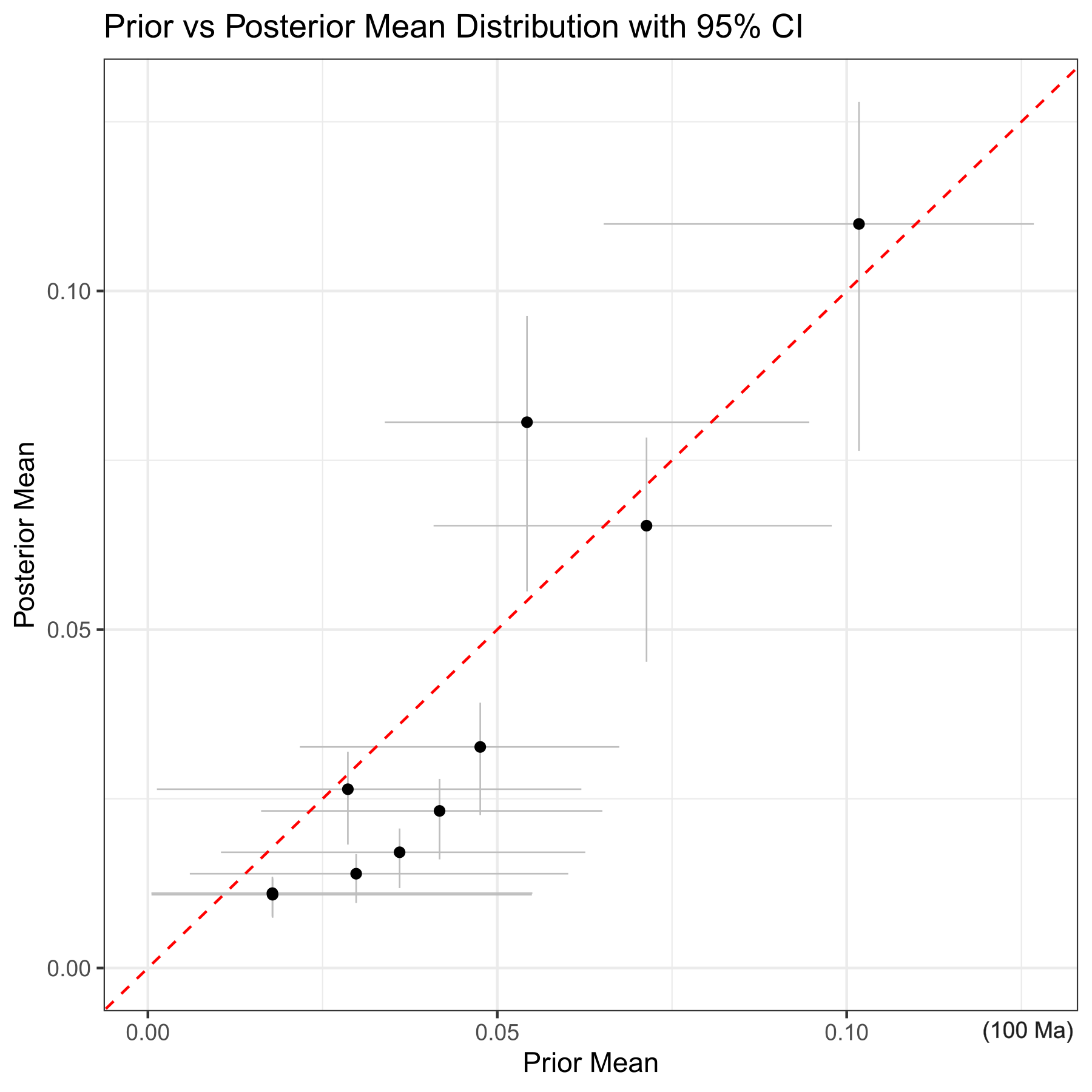


**Figure S2** Prior–posterior plots for node age mean distributions of MCMCTree. Each point represents a node in the phylogeny with its 95% CI (grey line). (Time unit:

100 Ma)


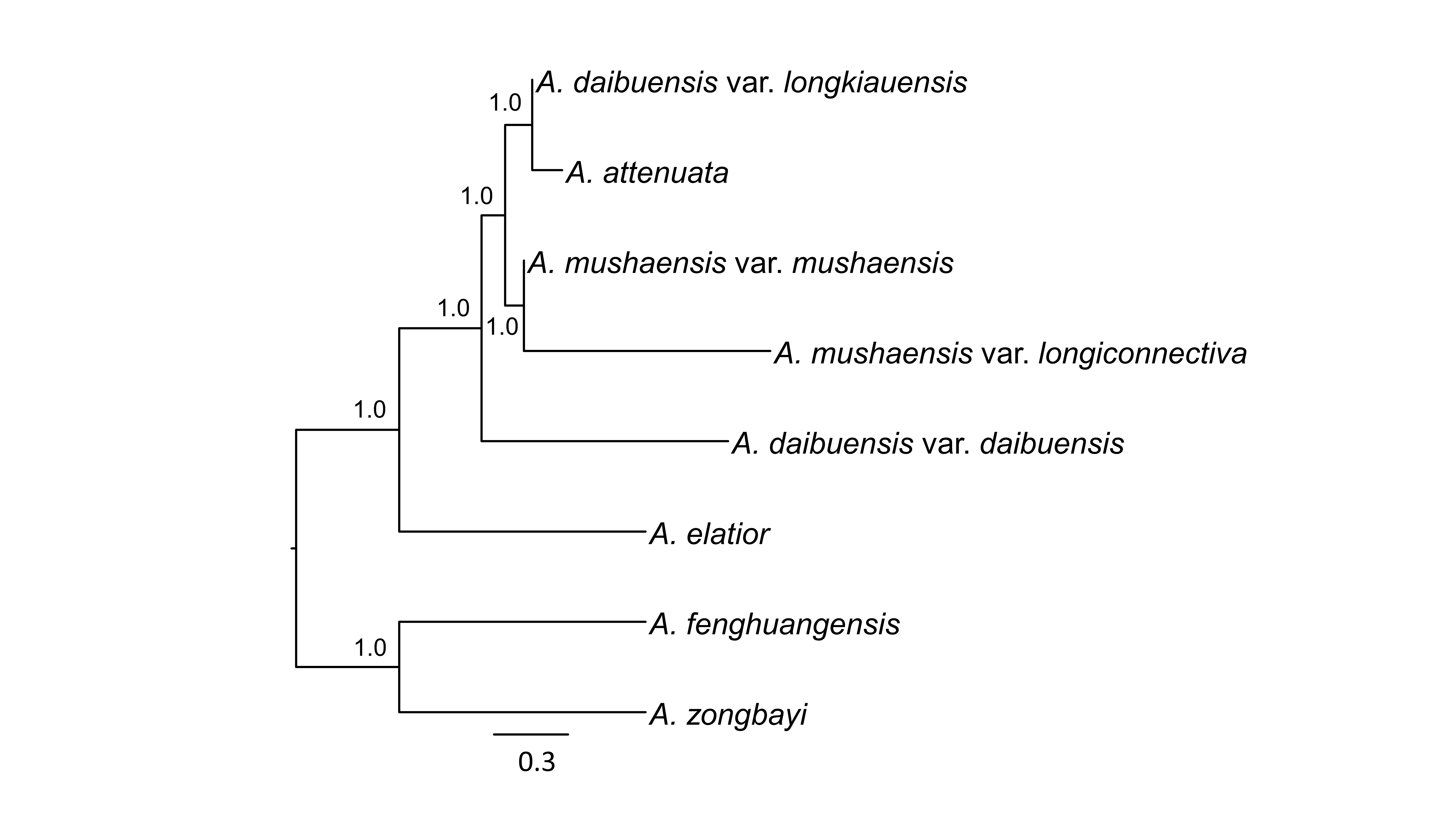


**Figure S3** Species tree reconstructed by ASTRAL with 1,029 genes. Node number indicates local posterior support score.
